# A Diary Study on Anticipated Leisure Time, Morning Recovery, and Employees’ Work Engagement

**DOI:** 10.3390/ijerph18189436

**Published:** 2021-09-07

**Authors:** Sebastian Seibel, Judith Volmer

**Affiliations:** 1 Work and Organizational Psychology Group, Otto-Friedrich University of Bamberg, 96047 Bamberg, Germany; judith.volmer@uni-bamberg.de; 2 Work and Organizational Psychology Group, Julius Maximilian University of Würzburg, 97070 Würzburg, Germany

**Keywords:** morning recovery, leisure time, pleasant anticipation, work engagement, thoughts of a planned leisure activity, diary study

## Abstract

Recovery during yesterday’s leisure time is beneficial for morning recovery, and morning recovery fosters employees’ work engagement, a positive, motivational state associated with job performance. We extended existing research by assuming that both, morning recovery (considered a resource) and anticipated leisure time (considered an anticipated resource gain), relate to work engagement. Anticipated leisure time comprises two constructs: general anticipation of leisure time, which refers to employees’ cognitive evaluation of their entire upcoming leisure time, and pleasant anticipation of a planned leisure activity, which describes a positive affective reaction because of one specific, upcoming leisure activity. We suggested that employees with high pleasant anticipation generate more thoughts of a planned leisure activity (ToPLA), which may distract them from their work, reducing their work engagement. A diary study over five days showed that morning recovery and general anticipation of leisure time were positively related to work engagement. Furthermore, employees with higher pleasant anticipation of a planned leisure activity reported more ToPLA. In contrast to our expectations, neither pleasant anticipation nor ToPLA was related to work engagement. In sum, this study introduced anticipated leisure time as a novel antecedent of work engagement and demonstrated that anticipated resource gains are important for high work engagement.

## 1. Introduction

During the working day, employees must deal with many different job demands and stressors, which drain their resources and exhaust their energy [1,2]. To stay healthy, recovery after the working day is essential for employees (for a meta-analysis, see [3]), and research has shown that leisure activities are critical to a successful recovery process (for a review, see [4]). Recovered employees are less fatigued and more vigorous (for a meta-analysis, see [5]) and report higher psychological well-being and job performance [3]. One indicator of job performance is employees’ work engagement [6,7], a positive, motivational, work-related state [8]. Work engagement is related to employees’ job performance and health (for a meta-analysis, see [9]); therefore, it is relevant for organizations and employees.

Until now, the majority of studies on recovery processes and work engagement have focussed on the relationship between yesterday’s leisure time and present work engagement e.g., [10,11,12]. For instance, participating in social activities during leisure time (e.g., meeting with friends) helps employees become more engaged during the next working day [12]. However, besides the importance of past leisure time, thinking about upcoming leisure time is part of everyday thoughts [13,14], and therefore, employees may also *anticipate* their upcoming leisure time while they are still at work. Previous research demonstrated that anticipation is relevant for employees’ behaviours, e.g., when employees anticipate a high workload, they put more effort into approach coping (e.g., planning their workdays; [15]). Furthermore, anticipated time pressure was shown to impair task performance in several experiments [16]. In sum, there is initial evidence that employees think about their leisure time and consider anticipated events. Thus, we extend the perspective of existent research on yesterday’s leisure time and investigate whether the anticipation of upcoming leisure time also relates to work engagement.

In our study, we take a closer look at two different types of anticipation of leisure time. First, we examine the general anticipation of leisure time, which refers to an overall evaluation of employees’ upcoming leisure time. Second, we investigate employees’ pleasant anticipation of a planned leisure activity, reflecting a positive, affective reaction because of one specific upcoming leisure activity (The term leisure activity is used for all activities that take place during employees’ leisure time). We draw on Conservation of Resources (COR) theory [17] and propose that both types of anticipation indicate an upcoming resource gain. According to COR theory [17], not only present but also anticipated resource gains and losses are part of individuals’ resource management [18,19]; therefore, we hypothesize that general anticipation and pleasant anticipation are positively related to work engagement. However, employees with higher pleasant anticipation of a planned leisure activity reported more thoughts of a planned leisure activity (ToPLA) during the working day [14]. ToPLA are a type of off-task thoughts (e.g., [20]), and research has shown that ToPLA are negatively related to work engagement [14]. Consequently, a negative relationship between pleasant anticipation of a planned leisure activity and work engagement, mediated by ToPLA, may also exist.

This study has three main research goals. First, we investigate whether the anticipation of upcoming leisure time and present work engagement are related. We thereby add to previous research on anticipation in the working context [15,16,21]. Because the anticipation of upcoming events plays an important role in individuals’ behaviour, feelings, and cognitions (e.g., [22,23]), and leisure time is a recursive part of employees’ daily lives, the current research aims at investigating further precursors of employees’ work engagement. Because work engagement has a substantial impact on employees’ well-being and performance [7,9], researchers are interested in identifying various antecedents of work engagement. These insights may point out new employee training programmes and add to existing recovery training [24].

The second aim of our study is to shed light on whether widely investigated previous leisure time experiences and anticipated leisure time activities are jointly associated with work engagement. Therefore, we refer to morning recovery as an indicator of a successful recovery process during yesterday’s leisure time and aim at replicating past research, demonstrating positive relationships between morning recovery and work engagement (e.g., [10,11,12]). Thus, we try to show that anticipated leisure time and yesterday’s leisure time (i.e., morning recovery) account for unique parts in work engagement.

**Figure 1:**
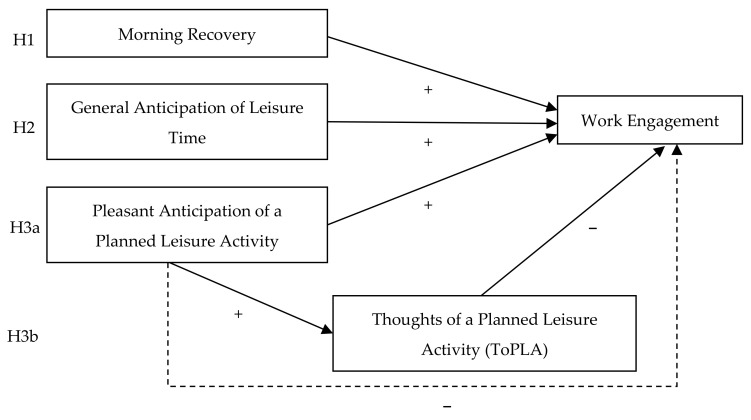
Research model. The dotted line represents the indirect effect through ToPLA.

Finally, because research on upcoming leisure time and work engagement has been limited to one working day and one planned leisure activity per participant [14], we followed two steps to extend the existing research. First, we collect several leisure activities per participant using a daily diary approach over five days to check for within-person differences in pleasant anticipation. We further investigate ToPLA as a within-person mediator between pleasant anticipation and work engagement. Second, by adding general anticipation of leisure time, we refer to overall leisure time and consider that employees’ leisure time contains more than just one leisure activity. Combining a diary study with two indicators of upcoming leisure time offers a comprehensive view of upcoming leisure time and helps to understand the mechanism that may link upcoming leisure time and present work engagement. Our research model is summarized in Figure 1.

### 1.1. Morning Recovery and Work Engagement

*Recovery* describes the process of unwinding from work-related stress when employees are free of work-related demands [25]. Apart from within-day work breaks [2], engaging in leisure activities provides opportunities to detach from work, relax, master new challenges, and experience control, helping employees prevent fatigue and make them more energetic (i.e., recovered) in the morning (for a meta-analysis, see [5]). Thus, morning recovery indicates how successful the recovery process was during yesterday’s leisure time and represents a resource [2,26].

During the working day, employees manage their resources by deciding whether to invest or save resources. Resources are defined broadly as everything that has a value for individuals [17] and helps people reach their goals, e.g., social support, time away from work and recovery experiences [27]. According to COR theory [17], employees stop investing resources (e.g., into their work) and enter a defensive mode of behaviour after their currently available resources fall below a specific resource limit. We define this turning point of resource investment as the lower resource limit. Because employees possess more resources in the morning after a successful recovery process [28,29], they can invest more resources into their work before they reach their lower resource limit, stop investing and start saving resources. Thus, in line with other research (e.g., [10,11,12]), we propose that morning recovery is positively related to work engagement and formulate the following hypothesis:

**Hypothesis** **1** **(H1).** *Day-level morning recovery will be positively related to day-level work engagement*.

### 1.2. Anticipated Resource Gains

According to COR theory [17], not only present but also upcoming resources are relevant for resource management, and anticipated resource gains and losses may carry similar consequences as actual resource gains and losses [18,19]. Research has shown that, for instance, the threat of a resource loss (i.e., an anticipated resource loss) decreased performance in an experimental study [30]. Furthermore, the anticipation of less workload in the future (i.e., more time for potential resource gains) relates to lower strain in the present [21]. Thus, evidence exists that anticipated resource gains and losses are associated with employees’ current states and behaviours.

We concentrate on anticipated resource gains and assume that employees, who anticipate a resource gain, know that they will (re)gain resources soon and do not need to save (many) resources. In terms of COR theory [17], employees’ lower resource limit decreases in anticipation of a resource gain; therefore, they can invest more resources. While morning recovery directly *increases* employees’ currently available resources, we posit that upcoming resource gains *decrease* employees’ lower resource limit. Because leisure time and leisure activities provide opportunities to (re)gain resources, general anticipation of leisure time and pleasant anticipation of a planned leisure activity may indicate upcoming resource gains.

### 1.3. General Anticipation of Leisure Time

Leisure time describes a time free from work, which employees could spend on activies of their own choice [31,32]. Because employees do not need to invest further resources, they can replenish consumed resources (e.g., energy) and gain new resources (e.g., social support). Thus, employees may anticipate their entire leisure time as a source for potential resource gains. We define general anticipation of leisure time as employees’ overall cognitive evaluation of their upcoming leisure time and propose that the general anticipation of leisure time indicates an anticipated resource gain. This cognitive evaluation of leisure time may depend on leisure time and current circumstances at work. For instance, when time pressure is high, employees may report greater anticipation of leisure time, as their upcoming leisure time indicates a reduction in time pressure. This example illustrates that the general anticipation of leisure time is not tied to a specific leisure activity but rather is an overall evaluation that depends on the present working day and future opportunities to (re)gain resources. In summary, when general anticipation of leisure time is high, employees expect high resource gains during leisure time. Consequently, employees’ lower resource limit decreases, and they can invest more resources into their work engagement. Thus, we propose the following hypothesis:

**Hypothesis** **2** **(H2).** *Day-level general anticipation of leisure time will be positively related to employees’ day-level work engagement*.

### 1.4. Pleasant Anticipation of a Planned Leisure Activity

Besides looking forward to their leisure time in general, employees may experience pleasant anticipation of one specific, planned leisure activity. Pleasant anticipation is defined as a positive affective reaction (e.g., joy, excitement), which is experienced only because of a positive, future event with a high probability of occurrence [14]. For instance, employees experience joy in the present (affective reaction) when they expect that meeting up with friends (future event) after work at 18:00 (high probability of occurrence) will be fun (positive evaluation of the event). Research on pleasant anticipation is rare but has demonstrated that pleasant anticipation relates to positive affect in the present, even though the anticipated event will happen in the future [33,34].

We focus on pleasant anticipation of a planned leisure activity to cover anticipated resource gains from different planned leisure activities, as leisure activities provide different opportunities to (re)gain resources (for a review, see [4]). For instance, employees may regain energy resources during low-effort activities and social support during social activities. Pleasant anticipation combines different characteristics of the upcoming leisure activity (e.g., expectation of the outcome, probability of occurrence), and therefore, describes whether employees expect a high and probable resource gain from this leisure activity. We postulate that pleasant anticipation of a planned leisure activity indicates an upcoming resource gain, reducing employees’ lower resource limit and allowing them to invest more resources into their work engagement. Thus, we propose the following hypothesis:

**Hypothesis** **3a** **(H3a).** *Day-level pleasant anticipation of a planned leisure activity will be positively related to employees’ work engagement*.

### 1.5. Thoughts of a Planned Leisure Activity (ToPLA) as a Mediator

Research has demonstrated that employees with high pleasant anticipation of a planned leisure activity think more frequently about anticipated leisure activities [14], which may distract them from the task at hand, reducing their work engagement. These thoughts are referred to as thoughts of a planned leisure activity (ToPLA) and are defined as “future-oriented thoughts of a specific plan that employees have in mind for their leisure time” [14]. Pleasant anticipation is an antecedent of ToPLA for two reasons [14]: First, higher pleasant anticipation indicates a higher probability of occurrence for the planned leisure activity, i.e., employees have a more concrete cognitive representation of that leisure activity (see construal-level theory; [35,36]). Because of the more concrete mental representation, employees recognize more environmental cues referring to their leisure activity, and therefore, ToPLA are more likely to be retrieved from memory [37,38]. Second, as thoughts of positive (compared to negative) future events are generated more easily [39,40], high pleasant anticipation may also increase the frequency of ToPLA. Thus, we propose that pleasant anticipation is positively related to ToPLA.

During the working day, employees must remain focussed on their tasks to demonstrate high performance [20], and off-task thoughts are negatively related to performance (for a meta-analysis, see [41]). ToPLA are a special type of off-task thoughts that drive attention away from the task at hand [14]. Therefore, when employees generate more ToPLA, they may concentrate less on their work and may show lower work engagement. In sum, we posit an alternative hypothesis. Specifically, we hypothesize that the relationship between pleasant anticipation of a planned leisure activity and work engagement is negative and mediated through employees’ ToPLA:

**Hypothesis** **3b** **(H3b).** *Day-level ToPLA will mediate the negative relationship between day-level pleasant anticipation of a planned leisure activity work engagement*.

## 2. Method

### 2.1. Overview

We conducted an online diary study comprising a pre-survey and three daily questionnaires across five consecutive workdays (Monday–Friday). After registration, participants were asked to choose a typical working week for participation (i.e., a week with regular working hours and without vacations) and were invited to fill out a pre-survey. During the five days of the study, participants received emails with links to three different questionnaires: a morning questionnaire (available from 6:00–10:00), a noon questionnaire (available from 11:00–14:00) and an afternoon questionnaire (available from 16:00–19:00). Participants were instructed to respond to the morning questionnaire at the beginning of their working day and to the afternoon questionnaire at the end of their working day. Data collection started in September 2019 and ended in May 2020, with a break in December and January due to public holidays. The registration was completed by 175 employees, and 138 employees filled out the pre-survey (78.86%). Participants responded on average to 9.51 of 15 questionnaires (response rate: 63.40%), and we collected data from 1369 single measurement points.

### 2.2. Participants

We recruited participants in Germany via flyers, social media, and personal contacts. Furthermore, four students helped us with data collection as part of their master’s thesis. Participants were required to be over 18 and have regular working hours (i.e., between 6:00–19:00) for at least 20 h a week. Participation was voluntary, and participants could receive individual feedback on their work engagement and morning recovery and participate in a lottery (4 × 50 €). Demographic information was available for 134 employees. More than half of the participants were female (59.71%), and participants’ average age was 37.84 years (*SD* = 12.66; range: 18–63). Most of them held a master’s degree (48.51%), a bachelor’s degree (14.93%), or finished a vocational training programme (16.42%). Participants worked in a variety of occupational sectors, e.g., social and health care services (20.90%), other service activities (12.69%), and information and communication (10.45%). On average, they worked 41.49 h per week (*SD* = 6.83). Some participants had leadership positions (28.36%), and only a few were self-employed (7.46%).

### 2.3. Measures

In the pre-survey, we assessed the demographic variables. In the morning questionnaire, we asked about employees’ morning recovery. In the noon questionnaire, we measured general anticipation of leisure time and pleasant anticipation of a planned leisure activity. In the afternoon questionnaire, employees’ work engagement and their ToPLA were assessed. Unless otherwise stated, all questionnaires used a five-point Likert scale ranging from 1 (totally disagree) to 5 (totally agree). We followed the recommendations by Nezlek [42] for computing reliability in daily diary research. He recommends using formulas provided by Shrout and Lane [43], which take into account not only different measurement points, but also the time nested within persons (i.e., the variance of the items and the time). Thus, within-person reliability indicates generalizability of within-person time variations averaged over items, and between-person reliability indicates generalizability of between-person differences averaged over time. We used the R package psych [44] to conduct the reliability analyses.

#### 2.3.1. Morning Recovery

Morning recovery was assessed with a scale developed by Sonnentag and Kruel [45]. The scale comprised four items, for example, “I feel mentally recovered”. Within-person reliability was 0.83, and between-person reliability was 0.78.

#### 2.3.2. General Anticipation of Leisure Time

To measure employees’ general anticipation of leisure time, we used a Kunin scale [46], which consisted of seven different smiley faces from 1 (picture of a very unhappy smiley face) to 7 (picture of a very happy smiley face). The instruction read: “Overall, how much are you looking forward to your upcoming leisure time?”. A similar measure was used previously to measure happiness during leisure activities [47]. Between-person reliability was 0.71, and because we used a single item measurement, within-person reliability could not be computed.

#### 2.3.3. Pleasant Anticipation of a Planned Leisure Activity

We first asked participants to describe a planned leisure activity in three words. They mentioned, for example, “reading a book”, “go shopping”, or “do sports”. Participants then were asked to classify their planned leisure activity into one of six categories (physical, social, low-effort, household, childcare, or work/administrative), a categorization schema commonly used in recovery research (e.g., [12,48]). Afterwards, we used a scale developed by Seibel et al. [14] to measure pleasant anticipation of a planned leisure activity with four items. A sample item was “I am looking forward to my leisure activity”. Within-person reliability was 0.84, and between-person reliability was 0.69.

#### 2.3.4. Work Engagement

We used the short version of the Utrecht Work Engagement Scale (UWES-9; [8]) to measure work engagement. We selected employees’ work engagement during the afternoon to refer to the period after the noon questionnaire. To address this period, we started each sentence with “In the afternoon” and removed three items, which did not fit into this period (e.g., “When I get up in the morning, I feel like going to work”). The scale comprised six items, e.g., “In the afternoon, I was immersed in my work”. Within-person reliability was 0.85, and between-person reliability was 0.84.

#### 2.3.5. Thoughts of a Planned Leisure Activity (ToPLA)

To measure employees’ ToPLA, we used a single item [14]: “How frequently did you think of the planned leisure activity (here, the planned leisure activity from the noon questionnaire was displayed) that you indicated in the last questionnaire?”. Participants could use an abstract rating scale from 1 (not at all) to 10 (all the time) to answer the question (i.e., participants should not indicate the concrete number of ToPLA). Between-person reliability was 0.71. Again, because we used a single item measurement, within-person reliability could not be computed.

## 3. Results

Our data was collected in a diary study (i.e., the same employee answered the same questionnaire several times); thus, the data comprised days (Level 1) nested within persons (Level 2), and we used a multilevel approach for our analyses [49]. Means, standard deviations, intraclass correlation coefficients (ICC) and correlations for all variables are presented in Table 1.

### 3.1. Planned Leisure Activities

We collected data from 449 different planned leisure activities. Participants classified 34.30% as physical activities, 26.73% as social activities, 22.72% as low-effort activities, 10.47% as household activities, 4.01% as work or administrative activities, and 0.67% as childcare activities (1.11% were not classified). Pleasant anticipation generally was high (*M* = 3.85, *SD* = 0.93), and a univariate analysis of variance (ANOVA) revealed a significant difference in pleasant anticipation depending on the type of leisure activity, *F*(1, 5) = 18.13, *p* < 0.001. Even though variances within the groups were equal, we did not conduct post-hoc contrast analyses because of the highly different group sizes. Descriptive analyses indicated that participants experienced low pleasant anticipation for planned administrative/work activities (*n* = 18, *M* = 2.65, *SD* = 1.09) and for planned household activities (*n* = 46, *M* = 3.12, *SD* = 0.93) and high pleasant anticipation for childcare (*n* = 3, *M* = 4.17, *SD* = 1.04), social activities (*n* = 119, *M* = 4.12, *SD* = 0.77), physical activities (*n* = 154, *M* = 3.98, *SD* = 0.79), and low-effort activities (*n* = 102, *M* = 3.91, *SD* = 0.86).

### 3.2. Hypotheses Testing

**Figure 2:**
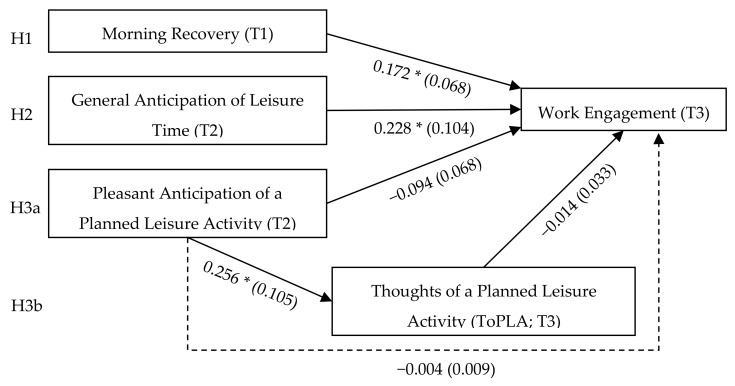
Results from multilevel path analysis. *N* = 110 employees; 302 matched questionnaires (T1 = morning, T2 = noon, and T3 = afternoon). Results show unstandardized estimates and standard errors in parentheses within persons. The dotted line represents the indirect effect through ToPLA. Model fit indices: *Χ*^2^ = 6.034, *df* = 4, Akaike information criterion = 4045.692, comparative fit index = 0.949, root mean square error of approximation = 0.041. * *p* < 0.05.

We used multilevel path modelling in Mplus Version 7.4 [50] to test all hypotheses in one model. A model with random intercept and fixed slopes was used for our analyses. To test the mediation, we followed the recommendations by Preacher et al. [51] and also modelled the mediation at Level 2 (2-2-2), although our main focus was on the lower-level mediation (1-1-1). The results for Level 1 are summarised in Figure 2, and the complete results from multilevel path modelling are presented in Table 2. Because the focus of our study was on daily changes within persons, we referred only to within-person effects when testing our hypotheses.

In Hypothesis 1, we assumed that daily morning recovery is positively related to daily work engagement. The path coefficient was significant within persons (estimate = 0.172, *SE* = 0.068, *p* = 0.011), supporting Hypothesis 1. In Hypothesis 2, we suggested that daily general anticipation of leisure time is positively related to daily work engagement. This relationship was significant within persons (estimate = 0.228, *SE* = 0.104, *p* = 0.028). Thus, Hypothesis 2 was supported.

For Hypotheses 3a,b, we proposed alternative hypotheses, positing that the relationship between day-level pleasant anticipation of a planned leisure activity and work engagement is either positive (H3a) or that the relationship is negative and mediated through employees’ ToPLA (H3b). The results showed a negative, non-significant relationship between pleasant anticipation and work engagement (estimate = −0.094, *SE* = 0.068, *p* = 0.171), rejecting Hypothesis 3a. Regarding Hypothesis 3b, pleasant anticipation of a planned leisure activity was positively related to ToPLA within persons (estimate = 0.256, *SE* = 0.105, *p* = 0.015). However, ToPLA was not related to work engagement within persons (estimate = −0.014, *SE* = 0.033, *p* = 0.675), and the indirect effect between pleasant anticipation and work engagement through ToPLA was not significant (indirect effect = −0.004, *SE* = 0.009, *p* = 0.681). Hence, Hypothesis 3b was rejected. At the between-person level, only the relationship between recovery and work engagement was significant (estimate = 0.407, *SE =* 0.169, *p* = 0.016).

## 4. Discussion

The overarching goal of our research was to answer the question of whether both upcoming leisure time and yesterday’s leisure time relate to work engagement. First, morning recovery (a consequence of yesterday’s leisure time) and work engagement were positively associated, concurring with a large amount of previous research (e.g., [10,11,12]). When employees started recovery in the working day, more resources were available (e.g., more energy), which boosted their work engagement. Second, daily general anticipation of leisure time related positively to work engagement, and employees who expected a positive, upcoming leisure time were more engaged in their work. In terms of COR theory [17], employees’ lower resource limit, which indicates the turning point of resource investment, decreased because of anticipated resource gains. Therefore, employees could invest more resources without the threat of investing more resources than they could (re)gain during their leisure time. Finally, we assumed that day-level pleasant anticipation of a planned leisure activity is related either positively or negatively to work engagement and that the negative relationship is mediated through ToPLA. In contrast to our expectations, the results from multilevel path modelling supported none of these hypotheses, and day-level pleasant anticipation was not related to work engagement. When employees looked forward to a specific, planned leisure activity, their work engagement did not change.

There are several explanations why pleasant anticipation was not related to work engagement. First, pleasant anticipation refers to only one planned leisure activity, and therefore, pleasant anticipation covers only one part of employees’ leisure time. While participants might report pleasant anticipation for a positive leisure activity, negative activities during leisure time might also exist, so overall work engagement did not increase. Because most employees mentioned a positive planned leisure activity when asked for pleasant anticipation, negative (i.e., resource-draining) leisure activities might have been neglected. Second, employees indicated many different leisure activities across the week, which addressed different resources (e.g., energy, social support), and we referred to pleasant anticipation to measure the anticipated resource gains from these different leisure activities. Because only some anticipated resource gains may relate to work engagement, the relationship between pleasant anticipation and work engagement might be non-significant. Third, one may also speculate that the resource management process depends on employees’ implicit theories about their resources (see implicit theories about willpower; [52]). While some employees may assume that leisure activities could replenish invested resources and benefit from pleasant anticipation, other employees may believe that resources could not be re(gain)ed. Thus, differences between persons may explain the non-significant relationship.

The non-significant relationship between pleasant anticipation of a planned leisure activity and work engagement points out the difference between pleasant anticipation and general anticipation of leisure time. In contrast to pleasant anticipation, general anticipation refers to participants entire leisure time, including all positive and negative activities and events in the upcoming leisure time. Therefore, the cognitive evaluation of these activities and events is an overall rating of the anticipated resource gains employees expect. We suppose that this overall rating impacts employees’ resource management more than the evaluation of single leisure activities.

Apart from the positive relationship between pleasant anticipation of a planned leisure activity and work engagement, we also argued that this relationship might be negative and mediated by employees’ ToPLA. Pleasant anticipation was positively related to ToPLA; thus, looking forward to a positive leisure activity triggered thoughts about that activity. However, ToPLA were not related to employees’ work engagement, and ToPLA did not meditate the relationship between pleasant anticipation and work engagement.

The positive relationship between pleasant anticipation and ToPLA corresponds to a previous study [14]; however, in contrast to our results, that study also revealed a negative and significant relationship between *hourly* ToPLA and *hourly* work engagement. The focus of our study on differences between days (not hours) may explain the non-significant relationship between ToPLA and work engagement. First, because we asked employees for the frequency of ToPLA in the afternoon, it could be difficult for them to estimate the frequency of ToPLA for the entire working day (compared to an hourly measurement of ToPLA). As a result, they might have underestimated the frequency of ToPLA. Second, we assessed ToPLA once a day, and each measurement of ToPLA referred to a different planned leisure activity. One may speculate that the content of ToPLA is more critical for work engagement than the frequency. For instance, the frequency of ToPLA could be the same on two days, but ToPLA refers to positive imaginations of the planned leisure activity on one day and doubts about the occurrence of the planned activity on the other. While the positive imaginations may enhance work engagement, the doubts may distract employees from their work.

In summary, we did not find a direct nor indirect relationship between pleasant anticipation and work engagement. Nevertheless, our study demonstrated that high pleasant anticipation of a planned leisure activity was related to more ToPLA. Thoughts about leisure time during work have been neglected until now (for an exception, see [14]), although much research on thoughts about work during leisure time exists (for a meta-analysis, see [53]). Thus, our research demonstrated that employees experience thoughts in both ways.

### 4.1. Theoretical Implications

Our study provided evidence that not only past but also anticipated resource gains are meaningful, which concurs with COR theory [18,19]. We demonstrated that employees take their anticipated leisure time into account while they are still at work and that employees’ daily general anticipation of leisure time is beneficial for daily work engagement. More specifically, employees’ daily work engagement depends on two different mechanisms: employees’ currently available resources (i.e., morning recovery), which they can directly invest into work engagement, and employees’ anticipated resource gains, which reduce their lower resource limit without providing additional resources. The two mechanisms carry different implications for resource management, and we propose to pay attention to this differentiation when using COR theory [17] to explain behaviour.

We also showed that morning recovery could be considered a resource in COR theory [17] as proposed by other researchers [2,26]. This finding highlights the importance of leisure time in general, as leisure time provides many opportunities to recover and (re)gain resources (e.g., [3,4]). Furthermore, the relationship between recovery and work engagement was significant at a between-person level, indicating that employees with a higher morning recovery over the working week were more engaged than employees with a lower morning recovery. Thus, average and day-level morning recovery are crucial for employees’ work engagement.

### 4.2. Practical Implications

Our study showed that the general anticipation of leisure time and morning recovery were associated with higher work engagement and revealed that pleasant anticipation of a planned leisure activity was related to ToPLA. Therefore, the practical implications are twofold: On the one hand, leisure time should include activities that foster recovery and resource replenishment to start the next working day with a high level of morning recovery (for a review, see [4]). These activities should be embedded in an overall positive leisure time such that employees could look forward to this upcoming leisure time and anticipate a resource gain. Even though leisure time may include negative leisure activities (e.g., cleaning chores, doing laundry), employees could enrich their leisure time with smaller positive components (e.g., calling a friend, listening to music).

On the other hand, specific planned leisure activities combined with high pleasant anticipation should be considered critical. ToPLA did not relate to work engagement, yet ToPLA are a type of off-task thoughts that can drive the attention away from the current task [41]. Thus, employees should try to reduce the frequency of ToPLA to stay concentrated at work, although ToPLA may not directly affect their work engagement. Therefore, we propose that employees plan their leisure activities in the morning, using a fixed time (e.g., five minutes) to concentrate on the planned leisure activity. Furthermore, to prevent a conflict between leisure and work-related plans, it may be beneficial when employees postpone their extraordinary leisure plans, which are more likely to evoke high pleasant anticipation, to the weekend.

### 4.3. Limitations and Future Research

We investigated general anticipation of leisure time as well as pleasant anticipation of a planned leisure activity, and both constructs were correlated. From a theoretical perspective, planned leisure activities are part of employees’ leisure time, and on days with high pleasant anticipation, general anticipation should also be high (explaining the within-person correlation). Furthermore, individuals differ in their ability to enjoy or savour future events [54], and individuals who can better enjoy their anticipated leisure time may also enjoy their planned leisure activities more (explaining the between-person correlation). From an empirical perspective, only the general anticipation of leisure time related positively to work engagement, demonstrating that differentiation between pleasant anticipation and general anticipation is necessary. In summary, we assume that both constructs are different, and we suggest that future research should consider them distinct.

To keep our daily questionnaires short and to enhance participants’ compliance [55], we used two single-item measurements throughout our study (Kunin scale for general anticipation, abstract rating scale for ToPLA). Between-person reliabilities for these items were acceptable, but we could not compute within-person reliabilities. However, within- and between-person reliabilities typically are not completely different (e.g., [56,57]), suggesting that the two single-item measurements may also be generalizable within persons. Furthermore, Kunin scales are commonly used to assess general evaluations, e.g., job satisfaction (for a meta-analysis, see [58]) or overall leader satisfaction [59]. Thus, we assume that the Kunin scale was appropriate to measure the general anticipation of leisure time. For ToPLA, future research could develop additional items based on questionnaires that measure work-related thoughts during leisure time (e.g., [60,61]).

We focused our research on employees’ subjective perception of their upcoming leisure time and activities. However, one may argue that upcoming leisure time indicates a reward after the working day and that neurophysiological processes of reward anticipation (e.g., [62,63]) may be important to understand the relationship between general anticipation of leisure time and work engagement. Thus, future research could use neuroscience methods [64] to further investigate the anticipation of leisure time.

Finally, all of our variables of interest referred to employees’ affective reactions (e.g., pleasant anticipation), states (work engagement), and thoughts (ToPLA) and were best accessible through self-ratings. To reduce common method variance [65], we measured our predictor variables and criterium variable at different measurement points and used different scale formats (Likert-scale, Kunin scale, abstract rating scale). Future research could expand our research on work engagement to outcomes rated by co-workers and supervisors to further decrease common method variance.

## 5. Conclusions

The present diary study demonstrated that morning recovery and general anticipation of leisure time are positively associated with work engagement. Thus, leisure time has two functions for employees at two different times. First, during the working day, leisure time indicates an upcoming resource gain. Second, after the working day, leisure time offers opportunities to realize this resource gain.

## Figures and Tables

**Table 1:** Means (*M*), standard deviations (*SD*), and correlations of the study variables.

		*M*	*SD*	ICC	1	2	3	4	5
1	Recovery (T1)	3.35	0.74	0.37		0.06	0.04	0.17 **	−0.02
2	General anticipation ^b^ (T2)	6.13	0.74	0.47	0.23 **		0.54 **	0.06	0.19 **
3	Pleasant anticipation ^a^ (T2)	3.81	0.68	0.29	0.14	0.66 **		0.06	0.20 **
4	Work engagement (T3)	2.93	0.76	0.48	0.26 **	0.12	0.08		−0.10
5	ToPLA (T3)	3.28	1.55	0.48	−0.10	0.19 *	0.23 *	−0.25 **	

Notes. T1 = morning; T2 = noon; T3 = afternoon; ToPLA = Thoughts of a planned leisure activity; ICC = intraclass correlation coefficient (percentage of variance between persons). Correlations below the diagonal are person-level (between-person) correlations (*N* = 113–133), and correlations above the diagonal are day-level (within-person) correlations (*N* = 307–447). ^a^ of a planned leisure activity. ^b^ of leisure time. * *p* < 0.05. ** *p* < 0.01.

**Table 2:** Results from multilevel path analyses.

Variables	Estimate	*SE*	*p*	95% CI
Within-person effects				
Recovery→Work engagement	0.172	0.068	0.011	[0.060; 0.284]
General anticipation ^b^→Work engagement	0.228	0.104	0.028	[0.057; 0.399]
Pleasant anticipation ^a^→Work engagement	−0.094	0.068	0.171	[−0.206; 0.019]
Pleasant anticipation ^a^→ToPLA	0.256	0.105	0.015	[0.082; 0.429]
ToPLA→Work engagement	−0.014	0.033	0.675	[−0.068; 0.040]
Pleasant anticipation ^a^→ToPLA →Work Engagement	−0.004	0.009	0.681	[−0.018; 0.011]
Between-person effects				
Recovery→Work engagement	0.407	0.169	0.016	[0.129; 0.686]
General anticipation ^b^→Work engagement	0.030	0.249	0.904	[−0.379; 0.439]
Pleasant anticipation ^a^→Work engagement	0.143	0.364	0.694	[−0.456; 0.742]
Pleasant anticipation ^a^→ToPLA	0.913	0.393	0.020	[0.266; 1.559]
ToPLA→Work engagement	−0.151	0.088	0.088	[−0.296; −0.006]
Pleasant anticipation ^a^→ToPLA →Work engagement	−0.138	0.100	0.166	[−0.302; 0.026]

Note. *N* = 110 employees; 302 matched questionnaires (morning, noon, and afternoon). Results show unstandardized estimates. Model fit indices: Χ^2^ = 6.034, *df* = 4, Akaike information criterion = 4045.692, comparative fit index = 0.949, root mean square error of approximation = 0.041. ^a^ of a planned leisure activity. ^b^ of leisure time.

## Data Availability

The data presented in this study are available on request from the corresponding author.
